# Reliability of ChatGPT answers to common questions on developmental dysplasia of the hip as an information source for parents

**DOI:** 10.3389/fped.2025.1659812

**Published:** 2025-12-15

**Authors:** Klemens Vertesich, Joachim Ortmayr, Reinhard Windhager, Madeleine Willegger

**Affiliations:** Department of Orthopedics and Trauma Surgery, Medical University of Vienna, Vienna, Austria

**Keywords:** developmental dysplasia of the hip, artificial intelligence, large language model, infant hip, hip screening

## Abstract

**Background:**

Artificial intelligence (AI), particularly AI-based large language models (LLM) like ChatGPT, is increasingly shaping how information is accessed, offering patients a new source for understanding complex medical conditions. Given the physical, emotional, and logistical challenges that parents are faced when their baby is diagnosed with developmental dysplasia of the hip (DDH), the demand for clear and accessible educational resources is high. This study aimed to evaluate the quality and reliability of ChatGPT's responses to frequently asked questions about DDH.

**Methods:**

This study assessed the quality of responses generated by the AI chatbot ChatGPT 4o to eight frequently asked questions about DDH, derived from real consultations in a pediatric orthopedic clinic Responses were generated during one interaction per question using a ChatGPT account not previously exposed to medical information. Responses were evaluated by two individual readers using a standardized rating system, comparing them to current literature, patient education resources, and consensus guidelines. Each response was categorized by its level of informational accuracy and completeness, and descriptive statistics were calculated to quantify performance.

**Results:**

ChatGPT 4o was able to generate structured responses to all eight parental questions. The responses were rated in 12.5% excellent, 25.0% satisfactory with minimal clarification, 50.0% satisfactory with moderate clarification, and 12.5% unsatisfactory due to missing or inaccurate information.

**Conclusion:**

ChatGPT provided satisfactory answers to questions about DDH and may serve as a useful supplementary information resource for parents. However, due to limitations in presenting detailed diagnostic and treatment pathways, it should be viewed as an adjunct to, not a replacement for, specialist medical consultation.

## 1 Introduction

Artificial intelligence (AI) has rapidly gained prominence in the medical field, continuously evolving and reshaping daily clinical practice (1). The development of AI-based large language models (LLM) such as ChatGPT has significantly transformed how information is accessed and utilized.

The internet has become a major source of medical information, with over 70% of patients routinely using it for health-related queries, and more than 80% of parents searching online for medical information concerning their children (2, 3). As the internet continues to serve as a primary platform for health education and decision-making, it is crucial for healthcare professionals, including surgeons, to remain up to date on emerging technologies that are influencing how patients obtain medical advice (4). In pediatric orthopedics, certain conditions can significantly affect the daily lives of patients and their families. Impairments such as reduced mobility, chronic pain, and limited joint function may not only disrupt physical activities but also contribute to psychological and emotional comorbidities (5).

Developmental dysplasia of the hip (DDH) represents one of the most common pediatric orthopedic conditions with an incidence of 1%–3% of infants (5), characterized by a broad clinical spectrum, ranging from acetabular dysplasia to complete dislocation of the femoral head. Typically diagnosed in the neonatal period through screening by clinical examinations, DDH can have lasting implications on mobility, function, and pain throughout childhood, adolescence, and even into adulthood if not appropriately managed (6). Management strategies for DDH, including the use of orthotic devices such as the Pavlik harness or spica casts, often place a substantial burden on caregivers, particularly during the early neonatal phase. These interventions may disrupt daily routines, require extensive compliance, and pose emotional and logistical challenges for families. Furthermore, the potential need for surgical correction in more severe or treatment-resistant cases can generate considerable anxiety and uncertainty among parents (6, 7). In such contexts, families frequently seek not only comprehensive medical guidance from healthcare professionals but also accessible, accurate, and comprehensible educational resources. The availability of reliable information becomes critical in helping parents understand the condition, adhere to treatment protocols, and participate actively in shared decision-making.

As AI tools become more integrated into healthcare communication, it is essential to assess their performance in delivering reliable, comprehensible, and patient-centered information. Several studies have assessed the accuracy, completeness, and clinical relevance of chatbot-generated responses to patient questions concerning common orthopedic procedures and conditions, including total hip arthroplasty (THA), slipped capital femoral epiphysis, and hip arthroscopy. These investigations have generally demonstrated that AI-generated content can provide satisfactory and informative answers, potentially supporting patient education and pre-consultation awareness (4, 8, 9). Further, the use of LLMs as assisting tools for investigation of pediatric diseases were described. Although results may look promising defining LLMs as additive tool, authors generally urge to caution quality inconsistencies and misinformation risks (10–12).

The aim of this study was to evaluate the quality, accuracy, and completeness of ChatGPT-generated responses to a set of frequently asked questions (FAQ) by parents regarding DDH. By comparing these responses with established patient education materials and current evidence-based literature, we aimed to assess the potential utility and limitations of ChatGPT as a supplementary tool for parents.

## 2 Methods

This study was designed as a quality assessment of responses generated by an AI chatbot. No patient or caregiver data were used, and ethical approval was not required due to the absence of human subjects. The study design was adapted from the methodology described by Mika et al. (8).

FAQs related to developmental DDH were identified and collected during consultations at our pediatric orthopedic outpatient clinic, which specializes in infant hip dysplasia and serves as a referral center for ultrasound-based screening of the infant hip. Questions were monitored over a time period of 6 months, from November 2024 to April 2025. Based on these observations, eight representative questions were formulated. These questions were formulated to represent a heterogeneity of clinically relevant issues in DDH. The questions were evaluated by all authors and revised upon further analysis. Questions were then submitted to ChatGPT 4o (OpenAI, San Francisco, CA, USA), with no follow-up or clarifying prompts provided. The used account was newly registered and was not previously exposed to orthopedic or medical questions. Questions were phrased and presented in English. The AI-generated responses were independently reviewed and evaluated by two pediatric orthopedic surgeons (K.V., consultant, and M.W., fellowship trained consultant). Inter-rater agreement was analyzed by using inter class correlation coefficient (ICC). After individual evaluation all answers were re-evaluated and definitive score was assigned upon agreement of all authors. Each response was assessed in comparison with current evidence-based literature. When applicable, responses were also evaluated against patient education resources and contemporary consensus statements from the Pediatric Orthopaedic Society of North America (POSNA), the European Federation of National Associations of Orthopaedics and Traumatology (EFORT), and peer-reviewed publications indexed in PubMed. Responses were rated according to the AI response rating system established by Mika et al., which categorizes output as follows: “excellent response requiring no further clarification,” “satisfactory response requiring minimal clarification,” “satisfactory response requiring moderate clarification,” and “unsatisfactory response requiring substantial clarification” (Table 1). For further analysis, results were stratified by the degree of informational deficiency: minimal (1–2 items omitted or requiring clarification), moderate (3–4 items), and unsatisfactory (more than 4 items omitted or requiring clarification, or inclusion of significant inaccuracies or misinformation). Numerical scores were assigned to each rating category. Descriptive statistical analyses and ICC were performed using SPSS version 29.0 (IBM Corp., Armonk, NY, USA).

**Table 1:** Overview on the used classification system published by Mika et al. (8).

ChatGPT Response Rating System	
Score	Response description/classification
1	Excellent response, requiring no further clarification
2	Satisfactory response, requiring minimal clarification
3	Satisfactory response, requiring moderate clarification
4	Unsatisfactory response, requiring substantial clarification

## 3 Results

ChatGPT 4o responded to all eight questions, generating answers structured in bullet-point format, typically ranging from three to seven items per response. Each response included subheadings with key points highlighted in bold, aiming to enhance readability and information retention for lay users. After analyzing the questions between the two readers there was an inter-rater agreement assessed by ICC of 0.93 (*p* < 0.001). Upon evaluation and agreement of the authors, the responses were classified as follows: one response was rated as excellent, requiring no further clarification; two responses were rated as satisfactory with minimal clarification; four responses were categorized as satisfactory with moderate clarification; and one response was deemed unsatisfactory, requiring substantial clarification due to omission of critical information and inclusion of misleading or inaccurate content. Table 2 provides an overview on classification of each question as well as a structured description and appraisal on readability for the targeted audience of each response.

**Table 2:** Overview on questions presented to ChatGPT 4o with classification and scoring of their response accuracy.

Question	Response accuracy scoring	Possible errors of responses	Readability
1. What is the likelihood of my infant developing hip dysplasia?	2	Lack in detailed information	Sufficient readability
2. Are there prenatal screening methods available for detecting developmental dysplasia of the hip?	1	No inaccuracy	Sufficient readability
3. How can developmental dysplasia of the hip be identified in my infant?	3	Outdated/not relevant information	Reduced readability/use of medical terms
4. What treatment options are available if my infant is diagnosed with developmental dysplasia of the hip?	3	Outdated/not relevant information	Reduced readability/use of medical terms
5. Will surgery be required if my infant is diagnosed with developmental hip dysplasia?	3	Outdated/not relevant information	Reduced readability/use of medical terms
6. How long does treatment typically last for developmental dysplasia of the hip?	2	Lack in detailed information	Reduced readability/use of medical terms
7. Are there potential long-term risks for my child if developmental dysplasia of the hip is diagnosed during infancy?	4	Incorrect and missing information	Sufficient readability
8. Is total hip arthroplasty likely to be necessary later in life if my child is diagnosed with developmental dysplasia of the hip as an infant?	3	Outdated/not relevant information	Sufficient readability
	Mean: 2.6		

In the following section, each question posed to ChatGPT 4o is presented alongside the corresponding AI-generated response and a detailed analysis is provided for each.

1. What is the likelihood of my infant developing hip dysplasia?
  - The likelihood depends on several factors:
    ○ **Family history:** If a close relative has had developmental dysplasia of the hip (DDH), the risk is higher.
    ○ **Breech presentation:** Babies born in a breech position (buttocks or feet first) are at higher risk.
    ○ **Gender:** Girls are more likely to develop DDH than boys.
    ○ **First-born children:** They have a slightly higher risk due to the tighter fit in the womb.
    ○ **Swaddling practices:** Tight swaddling that restricts leg movement can increase the risk.
    ○ The incidence of DDH ranges from **1 to 2 per 1,000 live births** for severe cases, though mild instability may be more common.

### 3.1 Answer analysis: satisfactory response, requiring minimal clarification

ChatGPT accurately identified several relevant factors that influence the incidence of DDH. It highlighted the increased risk associated with a positive family history, noting that first-degree relatives may have up to a 12-fold higher risk (13). The chatbot also addressed the mechanical theory related to DDH pathogenesis, correctly identifying breech positioning as a significant risk factor. However, other mechanical risk factors—such as oligohydramnios, macrosomia, and multiple pregnancy—were not mentioned (14, 15). Female sex, a critical risk factor, was correctly identified by the chatbot. Notably, up to 80% of DDH cases requiring treatment occur in females without any other associated risk factors. The chatbot also correctly identified being a first-born child as a risk factor for DDH (16). Cultural practices such as swaddling, which limit leg mobility and place the hips in extension and adduction, were recognized by the chatbot as significant contributors to DDH development (17, 18). However, associations between DDH and other congenital conditions, such as torticollis or clubfoot deformity, were not detected (14, 19, 20). The chatbot underestimated the incidence of clinically significant DDH requiring treatment, which is reported to range from 5 to 13 per 1,000 live births. Nonetheless, it did acknowledge the higher prevalence of milder forms of hip instability. Literature indicates that general hip instability is present in approximately 1%–1.5% of newborns, with nearly 90% of cases resolving spontaneously by eight weeks of age (21). The readably of the questions was sufficient and can be well interpreted by caregivers. Information were lacking in additional and detailed information and therefore we classified the results as satisfactory response, requiring minimal clarification.

1. Are there prenatal screening methods available for detecting developmental dysplasia of the hip?
  - Currently, there are **no reliable prenatal screening methods** to detect DDH.
  - Some risk factors, such as breech presentation or restricted fetal movement, may be identified via **prenatal ultrasounds**, but these are not definitive indicators of DDH.

### 3.2 Answer analysis: excellent response, requiring no further clarification

The chatbot accurately stated that, at present, no reliable method for prenatal screening of DDH is routinely used in clinical practice. It correctly described all clinically relevant and established diagnostic methods applicable during the neonatal period. However, recent studies have explored the feasibility of prenatal DDH screening, reporting promising results using an ultrasound technique modified after Graf around the 34th week of gestation (22–24). Although not yet integrated into routine clinical practice, these findings offer potential for future research and development in the field. Additionally, the chatbot appropriately noted that certain mechanical risk factors for DDH can be identified during the prenatal period (14, 15). The generated output was clear and easily understandable. Taken together, this response was considered to provide comprehensive and informative content for parents.

2. How can developmental dysplasia of the hip be identified in my infant?
  - DDH can be identified through:
    ○ **Physical exams:** Pediatricians perform hip stability tests such as the **Ortolani** and **Barlow maneuvers** during routine check-ups.
    ○ **Imaging:** Ultrasound is used for infants under 6 months, while x-rays may be used for older babies.
    ○ **Signs in your baby:** Asymmetrical thigh folds, a leg that appears shorter, or difficulty spreading the legs apart during diaper changes may indicate DDH.

### 3.3 Answer analysis: satisfactory response, requiring moderate clarification

ChatGPT correctly identified physical examination as a key component in the assessment of DDH. It referenced the Ortolani and Barlow maneuvers, which are routinely performed during the neonatal period. The Barlow maneuver involves adduction and posterior translation of the hip to provoke dislocation, whereas the Ortolani maneuver attempts to reduce a dislocated hip through abduction and anterior translation (21, 25). Additionally, ChatGPT recognized limited hip abduction as an important clinical sign of DDH. It also described a typical parental observation during diaper changes—difficulty abducting the hip—as a potential indicator of hip dislocation. This clinical finding is indeed characteristic of DDH and warrants further diagnostic evaluation (7, 26, 27). The chatbot also mentioned inguinal fold asymmetry, which, while associated with DDH, has limited diagnostic value, as it may appear in up to 30% of otherwise normal hips (28). Clinical examination alone may not be sufficient for diagnosing DDH. Notably, over 90% of individuals who underwent THA due to undetected DDH had inadequate screening during infancy (29). Regarding imaging, ChatGPT accurately identified it as a critical component of DDH evaluation. During the neonatal period—up to 4–6 months of age—ultrasound is the preferred imaging modality. The widely accepted Graf method assesses the bony and cartilaginous coverage of the femoral head by the acetabulum and is used universally as a screening tool in several countries (30). The method described by Terjesen et al. represents an alternative approach that incorporates dynamic parameters of hip development (31). he universal implementation of ultrasound screening remains a topic of ongoing debate. The European Society of Paediatric Radiology currently recommends ultrasound only for newborns with specific risk factors, such as breech presentation or a positive family history of DDH (15). After six months of age, when ossification of the femoral head has progressed, radiographs become the primary imaging method. However, due to concerns regarding radiation exposure, radiography is typically reserved for cases with a clinical suspicion of DDH rather than used for general screening purposes (32). While ChatGPT identified most key aspects of DDH assessment, several explanations lacked sufficient depth. Therefore, this response was classified as satisfactory, requiring moderate clarification.

3. What treatment options are available if my infant is diagnosed with developmental dysplasia of the hip?
  - Treatment depends on the severity and age of diagnosis:
    ○ **Pavlik harness:** A soft brace used to keep the hips in a stable position, typically for babies under 6 months old.
    ○ **Closed reduction:** A nonsurgical procedure to reposition the hip, followed by a cast (spica cast) to maintain alignment.
    ○ **Open reduction:** Surgical correction may be needed for more severe cases.
    ○ **Post-treatment monitoring:** Regular follow-ups with imaging to ensure proper hip development.

### 3.4 Answer analysis: satisfactory response, requiring moderate clarification

The primary goal in the treatment of DDH is to achieve a concentric and stable reduction of the femoral head within the acetabulum, while minimizing the risk of complications such as avascular necrosis (AVN) of the femoral head or re-dislocation (15). ChatGPT correctly identified the Pavlik harness as the most commonly used and important orthotic device for early treatment of DDH. It is generally considered the first-line therapy for infants with reducible hips (33). By positioning the hips in flexion and abduction, the harness facilitates spontaneous reduction of a dislocated hip. However, its effectiveness relies heavily on proper application and continuous use, requiring close cooperation from both patients and parents (34). The chatbot recognized the Pavlik harness as the preferred initial treatment method but did not elaborate on its limitations or contraindications. It also appropriately identified closed reduction followed by spica casting as a common next step in cases where conservative treatment fails, and acknowledged open reduction as a necessary intervention in more severe or unresponsive cases. However, it did not clearly explain the clinical indications that determine the transition from closed to open reduction. In practice, both closed and open reductions are performed under general anesthesia in the operating room. If closed reduction is unsuccessful—either due to failure to achieve a stable, concentric position of the femoral head or inconclusive findings on intraoperative arthrography—open reduction is indicated (35, 36). Open reduction is more frequently required in older children or when anatomical blocks to reduction prevent closed reduction (15). Follow-up assessments using plain radiographs, 3D radiographic techniques, or magnetic resonance imaging (MRI) are essential to confirm maintained reduction and to detect early signs of AVN (37, 38). The answers output was comprehensive. Treatment names such as Pavlik harness were not explained making it initially not fully understandable for persons without medical knowledge and definitely requires further follow-up questions and explanations. Taken together the response was classified as satisfactory, requiring moderate clarification.

4. Will surgery be required if my infant is diagnosed with developmental hip dysplasia?
  - Surgery is not always required:
    • Mild cases often resolve with noninvasive treatments like the **Pavlik harness**.
  - Surgery (closed or open reduction) is more likely if:
    ○ The diagnosis is delayed.
    ○ Noninvasive treatments are unsuccessful.
    ○ The hip is severely displaced.

### 3.5 Answer analysis: satisfactory response, requiring moderate clarification

The chatbot correctly stated that surgical intervention is not always necessary in the management of DDH. As outlined in the previous section, the Pavlik harness is considered the first-line treatment in the majority of DDH cases, with many pathological changes resolving within the first six weeks of life (39). Reported success rates for the Pavlik harness range between 85% and 92% (40). When non-surgical management fails, treatment must be escalated to closed or open reduction (15, 41). The chatbot also identified unsuccessful non-operative treatment and delayed or late diagnosis as risk factors for surgical intervention. This is consistent with current literature, which links delayed treatment to an increased likelihood of requiring multiple surgical procedures (42). However, the chatbot did not elaborate on the age-related risks associated with surgical treatment, such as the potential need for acetabular interventions in older children. Studies report that up to 19% of patients who initially respond well to Pavlik harness therapy may later exhibit residual acetabular dysplasia, necessitating additional surgical procedures—most commonly acetabular osteotomies—during childhood (43–45). While the chatbot successfully identified general factors that may lead to surgical intervention, providing a useful overview for parents, it lacked coherence in explaining the clinical decision-making process regarding when surgery becomes necessary. Therefore, this response was classified as satisfactory, requiring moderate clarification.

5. How long does treatment typically last for developmental dysplasia of the hip?
  - Treatment duration varies:
    ○ **Pavlik harness:** Typically worn for 6–12 weeks, depending on progress.
    ○ **Spica cast:** May be needed for 3–6 months after closed or open reduction.
    ○ Regular monitoring may continue for **several years** to ensure normal hip development.

### 3.6 Answer analysis: satisfactory response, requiring minimal clarification

ChatGPT successfully identified variations in treatment duration for DDH. It accurately reported the typical duration of Pavlik harness therapy as ranging from 6 to 12 weeks (7, 46). However, in cases with suboptimal response to treatment, prolonged use may be necessary, and escalation to surgical intervention may be required. The chatbot also correctly stated that the duration of spica casting following closed or open reduction is generally around three months. However, it did not elaborate on the fact that treatment duration may be extended based on radiographic findings during follow-up (36). Additionally, ChatGPT recognized the importance of continued clinical and radiographic monitoring following initial treatment. Long-term follow-up is essential to ensure proper hip development and to identify residual dysplasia or complications such as AVN. Overall, the response provided by the chatbot was relatively accurate and informative, though it required minimal clarification. The answer was understandably phrased. The use of medical terms for immobilization requires follow-up explanation or further search for caregivers. Therefore, it was classified as satisfactory, requiring minimal clarification.

6. Are there potential long-term risks for my child if developmental dysplasia of the hip is diagnosed during infancy?
  - If treated early, most children develop normal hip function with minimal long-term risks.
  - Without treatment, DDH can lead to:
    ○ Hip pain or arthritis in adolescence or adulthood.
    ○ Limping or uneven leg lengths.
    ○ Decreased range of motion.

### 3.7 Answer analysis: unsatisfactory response, requiring substantial clarification

ChatGPT correctly identified that early and adequate treatment is key for normal hip development and function (6, 47, 48). Under 5% of suspicious findings on ultrasound persist or worsen during treatment (46). In the second part of its response, the chatbot mentioned potential long-term symptoms such as hip pain, leg-length discrepancy, and reduced range of motion. However, it did not clearly specify the timeframe in which these complications may arise. Despite sufficient early treatment, some patients with DDH may continue to experience hip instability, which can impair mobility and cause pain beginning in childhood or adolescence (44, 49). In such cases, surgical intervention, such as femoral osteotomies, may be indicated to reorient the femoral head, improve joint stability, and stimulate proper acetabular development (45, 50). Acetabular osteotomies are required to enhance acetabular coverage (15). Acetabuloplasties reshape the morphology of the cup and are used in younger patients where the acetabular growth plate has not yet ossified. More complex procedures, such as periacetabular osteotomy, may be necessary to achieve adequate acetabular reorientation and joint congruity (51). Age and skeletal maturity are critical factors that influence the choice and timing of surgical interventions. In cases of residual DDH, surgical treatment can significantly improve long-term outcomes. However, the optimal timing of such procedures remains a topic of ongoing debate in the literature (6, 40). While ChatGPT mentioned possible long-term symptoms, it failed to address the potential need for additional surgical treatments over the course of the disease. This represents a significant omission, particularly in the context of providing comprehensive information to parents. Therefore, the response was classified as unsatisfactory, requiring substantial clarification.

7. Is total hip arthroplasty likely to be necessary later in life if my child is diagnosed with developmental dysplasia of the hip as an infant?
  - Early diagnosis and treatment significantly reduce the likelihood of needing a total hip arthroplasty (hip replacement) later in life.
  - If untreated or if treatment is delayed, the risk of early-onset osteoarthritis increases, which may necessitate hip replacement in adulthood.

### 3.8 Answer analysis: satisfactory response requiring moderate clarification

The chatbot clearly recognized the importance of early and adequate diagnosis and treatment of DDH in reducing the risk of THA later in life. It accurately stated that undiagnosed or inadequately treated DDH can result in gait abnormalities, restricted range of motion, and chronic pain due to the development of osteoarthritis (52). By disrupting normal hip biomechanics, untreated hip dysplasia can accelerate the degeneration of both femoral and acetabular cartilage, ultimately leading to secondary osteoarthritis. Literature indicates that approximately 25%–50% of patients with persistent DDH develop radiographic or symptomatic signs of osteoarthritis by the age of 50, often requiring THA due to pain and functional impairment (53, 54). While the chatbot identified the key consequences of untreated DDH, including the long-term risk of osteoarthritis and subsequent surgical intervention, the response lacked depth and omitted additional context that would be valuable for informing and reassuring parents. Despite that the phrased answer was easily readable and understandable. Therefore, although the main points were addressed, the response could have been strengthened with more comprehensive detail.

## 4 Discussion

The LLM ChatGPT 4o demonstrated the ability to generate accurate and relevant responses to critical parental questions concerning DDH. Most of the responses required only minimal or moderate clarification. However, when compared with expert medical advice and critical appraisal of current literature, gaps in the depth and specificity of information became evident. Given the complexity and evolving nature of DDH diagnosis and treatment algorithms, fully capturing the nuance of clinical decision-making remains a challenge for AI systems. ChatGPT showed potential in delivering useful information to parents. It may provide an overview on complex pathologies like DDH to initially comprehend the disease and the necessity of treatment, while it cannot replace professional consultation. Further, the ability of parents to interpret AI-generated information varies widely and may be further impaired in emotionally stressful situations such as a DDH diagnosis. This creates clinical and ethical concerns while incomplete or overly reassuring responses could delay medical consultation, reinforce misconceptions, or undermine trust in healthcare professionals. Final treatment indication and detailed explanation in each individual case cannot be replaced by LLMs. ChatGPT may serve as a valuable initial source of information, helping to prepare patients and caregivers for more informed discussions with healthcare providers.

The use of AI-generated medical content, particularly through chatbots such as ChatGPT, has garnered growing interest in the medical, and especially orthopedic community. As AI tools rapidly evolve, they are transforming how patients and caregivers access health information, which in turn may influence healthcare-seeking behavior and treatment decisions (55). The ability of LLMs to process and communicate medical information has been supported by recent publications across a range of orthopedic conditions (4, 8, 9, 56–59). In this study, ChatGPT provided generally sufficient and helpful answers to FAQs about DDH. However, given the continuous development of AI models and the potential for variability in responses across different interactions, it is essential that AI-generated medical information be reviewed and verified by qualified healthcare professionals (4, 8).

Several limitations of this study must be acknowledged. The study design incorporated inherently subjective elements, including the selection and formulation of FAQs from parents, as well as the observation and documentation of the AI-driven conversation process. Additionally, the evaluation and classification of the chatbot responses were based on the authors' interpretation, which may introduce bias despite efforts to maintain objectivity. To enhance transparency and reproducibility, all AI-generated outputs were documented and shared. Furthermore, in order to minimize variability and improve internal validity, each question was submitted to the chatbot a single time. While this approach reduces the impact of response variation, it must be noted that repeated queries can yield slightly different responses, even if the core content and intent remain consistent. Additionally, responses of LLMs may vary in terms of reproducibility and generalizability limiting the results regarding these parameters. The study was conducted using ChatGPT-4o, which, at the time of inquiry, represented the most advanced version of the model available. Currently there is a more recent version, ChatGPT 5, available. However, ChatGPT does not possess continuous access to the internet and is limited by a training data cutoff of April 2024. Consequently, the chatbot may not reflect the most recent literature or clinical guidelines published thereafter. Importantly, ChatGPT was not assigned a specific role, for example physician or parent, during the interaction, nor was it provided with contextual information about the target audience. This approach was chosen to simulate a typical, unscripted interaction between users and the AI, as might occur in real-world usage. Nevertheless, the absence of such framing could have influenced the style and depth of responses, potentially affecting their classification.

## 5 Conclusion

ChatGPT frequently provided satisfactory responses to parental questions related to DDH. Based on the findings of this study, it may serve as a helpful supplementary source of information for parents confronted with a DDH diagnosis in their newborn child. However, the chatbot did not consistently present structured diagnostic pathways or detailed treatment algorithms, and most responses required some degree of clarification or supplementation. As such, AI-based chatbots should be regarded as adjunct tools rather than standalone sources of medical advice. In clinical practice, these tools may complement—but not replace—comprehensive consultation with a pediatric orthopedic specialist. Rapid developments of LLMs however may quickly lead to an implication and use of these models in clinical practice. Therefore continued development, validation, and oversight will be essential to ensure the safe and effective integration of AI-driven technologies into clinical practice as well as parent and patient education. LLMs will be useful and essential tool in future clinical practice and ongoing research, improvement and validation of these models is essential to further improve patient specific treatments.

## Data Availability

The raw data supporting the conclusions of this article will be made available by the authors, without undue reservation.
